# Transfer Learning Based Fault Diagnosis with Missing Data Due to Multi-Rate Sampling

**DOI:** 10.3390/s19081826

**Published:** 2019-04-17

**Authors:** Danmin Chen, Shuai Yang, Funa Zhou

**Affiliations:** 1 State Key Laboratory of Mathematical Engineering and Advanced Computing, Zhengzhou 450001, China; 10250087@vip.henu.edu.cn; 2 School of Software, Henan University, Kaifeng 475004, China; 3 School of Computer and Information Engineering, Henan University, Kaifeng 475004, China; 104753170694@vip.henu.edu.cn; 4 Department of Electrical Automation, Shanghai Maritime University, Shanghai 201306, China

**Keywords:** fault diagnosis, DNN, transfer learning, missing data

## Abstract

Deep learning is an effective feature extraction method widely applied in fault diagnosis fields since it can extract fault features potentially involved in multi-sensor data. But different sensors equipped in the system may sample data at different sampling rates, which will inevitably result in a problem that a very small number of samples with a complete structure can be used for deep learning since the input of a deep neural network (DNN) is required to be a structurally complete sample. On the other hand, a large number of samples are required to ensure the efficiency of deep learning based fault diagnosis methods. To solve the problem that a structurally complete sample size is too small, this paper proposes a fault diagnosis framework of missing data based on transfer learning which makes full use of a large number of structurally incomplete samples. By designing suitable transfer learning mechanisms, extra useful fault features can be extracted to improve the accuracy of fault diagnosis based simply on structural complete samples. Thus, online fault diagnosis, as well as an offline learning scheme based on deep learning of multi-rate sampling data, can be developed. The efficiency of the proposed method is demonstrated by utilizing data collected from the QPZZ- II rotating machinery vibration experimental platform system.

## 1. Introduction

The structure of automation equipment is becoming more and more complex. Once a component fails, the whole system will be paralyzed. Therefore, fault diagnosis has received increasing attention [1,2,3,4,5,6,7,8,9,10,11,12,13,14,15,16,17,18,19,20,21,22,23,24,25,26,27,28,29,30,31,32]. In general, three fault diagnosis methods are now taken into consideration by scholars: fault diagnosis methods based on a physical model [1,2], fault diagnosis methods based on knowledge [3,4,5,6] and data-driven methods [7,8,9,10]. Model-based methods require an accurate analytical model, which limits its application in the field of fault diagnosis [9,10]. Fault diagnosis methods based on knowledge rely on artificial experience. Because of the complexity of the system and the limitation of expert experience, it is hard to ensure the accuracy of knowledge-based diagnosis [10]. In contrast, data-driven methods rely on neither expert experience nor accurate physical model. Data-driven methods can obtain useful information by data mining technologies and have become practical diagnosis technologies at present [11,12,13].

In recent years, deep learning methods have grown rapidly in academia and industry as a kind of data-driven methods. Deep learning methods are widely used in fault diagnosis, such as deep belief networks (DBN) [14,15,16,17,18], stacked auto-encoders (SAE) [14,19,20,21,22,23,24], long short-term memory neural networks (LSTM) [25] and convolutional neural networks (CNN) [26,27]. However, despite the marvellous success of deep learning methods, the above proposed methods have great limitations: structurally complete samples are used for data analysis, feature extraction and fault diagnosis. The accuracy of fault diagnosis methods based on deep learning depends on the quantity and quality of samples. In practical engineering, the sampling rates of sensors are different, resulting in a small number of samples with complete structure and a large amount of missing data. Missing data means that there are one or more incomplete data for the observed variables in a database [33]. Random data packet dropout in the network, the multi-rate sampling of sensors, sensor failure and other reasons would lead to the phenomenon of missing data. For example, if there are two sensors with the sampling frequency of one ten times that of another, then structurally complete samples which have two sensor data at the same time account for only 10%. That is to say, 90% of the data is missing. Missing data will inevitably affect the accuracy of fault feature extraction, which cannot guarantee the accuracy of the fault diagnosis model and the validity of diagnosis methods. Reference [34] demonstrated that when there was not much training data, deep neural network (DNN) models may perform worse than other shallow models.

To address the problem of missing data, there are two common strategies: listwise deletion and imputation [33]. The listwise deletion removes all data for a case that has one or more missing values. The deletion is thus always the last choice, for it may lead to significant information loss. Data imputation includes mean substitution, regression substitution and K-nearest neighbour substitution. Mean substitution is an approach to fill the missing values by calculating the complete data mean. Mean substitution is mainly suitable for data sets with a normal distribution. Regression substitution establishes regression equations for missing attributes and other non-missing attributes to fill the missing values of missing attributes. The establishment of regression models depends on the linear correlation between attributes but there may not be a linear relationship between attributes. K-nearest neighbour substitution is to find K instances nearest to incomplete instance objects in complete data sets to fill missing attributes. Because each filling needs to traverse the instance space, dimensional disasters easily occur for large data sets. The filling method therefore has its own limitations as well. It utilizes the information of complete data sets so it can only be applied to data sets with random missing and small missing proportion. Moreover, because missing data can be filled at first and then fault diagnosis can be carried out, the imputation method cannot be used for real-time online fault diagnosis. When the proportion of missing data is relatively high, the performance of data imputation is intolerable.

The sampling rates of sensors are different resulting in a very small number of samples with complete structure and a large amount of missing data. A small number of complete samples cannot ensure the efficiency of deep learning based fault diagnosis methods. A large number of missing data is not suitable for data imputation. So this paper uses transfer learning to make full use of a large amount of missing data to improve the accuracy of fault diagnosis. Transfer learning aims to recognize and apply knowledge learned from previous tasks to novel tasks [35,36] and has made great progress in the areas of images [37,38,39], natural language processing [40,41] and medical health [42,43,44]. Reference [45] presented a multitask fuzzy system modelling method, which makes the most of the independent information of each task and correlation information captured by the common hidden structure among all tasks. Reference [46] proposed both online and offline weighted adaptation regularization algorithms to minimize the amount of labeled subject specific EEG data in BCI calibration in order to improve the utility of BCI system. Reference [47] presented a TL-SSL-TSK model which combines transfer learning, semi-supervised learning, and TSK fuzzy system models to enhance the robustness, accuracy and interpretability of EEG signal classifier. However, the research of transfer learning in the field of fault diagnosis is little yet [29,30,31,32]. Reference [29] proposed a deep transfer network based on a domain adaptation method for fault diagnosis but it only considers marginal distribution without taking into account conditional distribution. Reference [30] presented a fault diagnosis framework with joint distribution adaptation which can decrease the discrepancy in both marginal distribution and conditional distribution. Reference [31] proposed a transfer learning method for gearbox fault diagnosis based on a convolutional neural network. The proposed transfer learning architecture consists of two parts: the first part is constructed with a pre-trained convolutional neural network that serves to extract the features automatically from natural images; the second part trains the full connection layer by using gearbox fault experiment data. Reference [32] presented a transfer learning approach to fault diagnosis with a neural network in a variety of working conditions. Missing data is an important issue but existing articles do not deal with fault diagnosis of missing data based on transfer learning. In the paper, we present a transfer learning framework for fault diagnosis of missing data. A detailed comparison between References [29,30,31,32,46,47] and this paper is shown in Table 1.

In this paper, we propose a fault diagnosis framework of missing data based on transfer learning. Structurally incomplete samples may lose some key information but contain other useful information. It is necessary to transfer them to the structurally complete fault diagnosis model. In turn, the number of structurally complete samples is small but structurally complete samples contain all the information monitored. Therefore, the data with a complete structure are also transferred to the model with missing data to optimize fault diagnosis performance of missing data.

The remainder of this paper is organized as follows: Section 2 provides a literature review of deep neural networks. Section 3 describes a fault diagnosis framework with missing data based on transfer learning. In Section 4, the validity of the proposed fault diagnosis method is verified through a case study. Finally, the main conclusions are provided in Section 5.

## 2. Review of Deep Neural Network

A deep neural network (DNN) can be composed of multiple automatic encoders. It uses bottom-up unsupervised learning to extract features layer by layer and uses a supervised learning method to fine-tune the parameters of the whole network. DNN can extract the essential features from the original data. The autoencoder is a feedforward neural network which has an input layer, a hidden layer and an output layer. The output layer has the same number of nodes as the input layer in order to reconstruct its input and the hidden layer is taken as the learned feature. The autoencoder consists of encoding and decoding. The encoding is the mapping from the input layer to the hidden layer and the decoding is the mapping from the hidden layer to the output layer.

There is an unlabeled data set $\left\{x_{pm}\right\}$, (p=1,2,…,P;m=1,2,…,M) containing *P* variables and M samples. The encoding process is

(1) $$h_{m}=f_{\theta }(x_{m})=\sigma (Wx_{m}+b)$$

(2) $$\sigma (x)=\frac{2}{1+e^{-2x}}-1$$

where $f_{\theta }$ is encoding function, σ is the tansig activation function, W is the weight matrix between input layer and hidden layer, b is bias vector of encoding and θ={W,b} is a set of weight matrix and bias between input layer and hidden layer. Similarly, the decoding process is

(3) $$y_{m}=g_{\tilde{\theta }}(h_{m})=\sigma (\tilde{W}h_{m}+d)$$

where $g_{\tilde{\theta }}$ is decoding function, σ is the tansig activation function, $\tilde{W}$ is the weight matrix between hidden layer and output layer, d is bias vector of decoding and $\tilde{\theta }=\{\tilde{W},d\}$ is a set of weight matrix and bias between hidden layer and output layer.

The reconstruction error function $J_{\left(\theta ,\tilde{\theta }\right)}(x,y;W,b)$ is

(4) $$J_{\left(\theta ,\tilde{\theta }\right)}(x,y;W,b)=\frac{1}{m}||y-x|{|}^{2}$$

The aim of network training is to minimize the reconstruction error function $J_{\left(\theta ,\tilde{\theta }\right)}$ by gradient descent and back propagation. The updating rules of parameters are

(5) $$W=W-\alpha \frac{\partial }{\partial W}J_{\left(\theta ,\tilde{\theta }\right)}(x,y;W,b)$$

(6) $$b=b-\alpha \frac{\partial }{\partial b}J_{\left(\theta ,\tilde{\theta }\right)}(x,y;W,b)$$

(7) $$\tilde{W}=\tilde{W}-\alpha \frac{\partial }{\partial \tilde{W}}J_{\left(\theta ,\tilde{\theta }\right)}(x,y;\tilde{W},d)$$

(8) $$d=d-\alpha \frac{\partial }{\partial d}J_{\left(\theta ,\tilde{\theta }\right)}(x,y;\tilde{W},d)$$

In order to realize classification, this paper uses the Softmax classifier as the output layer of DNN. The training data set is $\left\{x_{m}\}(m=1,2,\ldots ,M\right)$. $u_{m}\in \{1,2,\ldots ,k\}$ is the label. The probability p(u=k|x) of each type $k^{}(k=1,2,\ldots ,K)$ can be calculated by the following hypothesis function,

(9) $$h_{\theta_{s}}(x_{m})=\left[\begin{array}{c} p(u_{m}=1|x_{m};\theta_{s})\\ p(u_{m}=2|x_{m};\theta_{s})\\ \vdots \\ p(u_{m}=k|x_{m};\theta_{s}) \end{array}\right]=\frac{1}{\sum_{k=1}^{K}e^{\theta_{sk}^{T}x_{m}}}\left[\begin{array}{c} e^{\theta_{s1}^{T}x_{m}}\\ e^{\theta_{s2}^{T}x_{m}}\\ \vdots \\ e^{\theta_{sK}^{T}x_{m}} \end{array}\right]$$

where $\theta_{s}$ is the parameter of Softmax. The loss function $J_{\theta_{s}}$ is

(10) $$J_{\theta_{s}}(x_{m})=-\frac{1}{M}[\sum_{m=1}^{M}\sum_{k=1}^{K}1\{u_{m}=k\}\log\frac{e^{\theta_{sk}^{T}x_{m}}}{\sum_{k=1}^{K}e^{\theta_{sk}^{T}x_{m}}}]$$

Finally, the DNN performs supervised fine-tuning by back propagation. The process of updating parameters can be written:

(11) $$E(\theta )=\frac{1}{M}\sum J_{\theta }(Y_{m},u_{m};\theta )$$

(12) $$\theta =\theta -\alpha \frac{\partial E(\theta )}{\partial \theta }$$

where $Y_{m}$ is predicted output, $\theta =\{\theta_{1},\theta_{2},\ldots ,\theta_{N},\theta_{s}\}$ is the set of parameters and is updated by back propagation algorithm.

## 3. A Fault Diagnosis Framework with Missing Data Based on Transfer Learning

**Figure 1:**
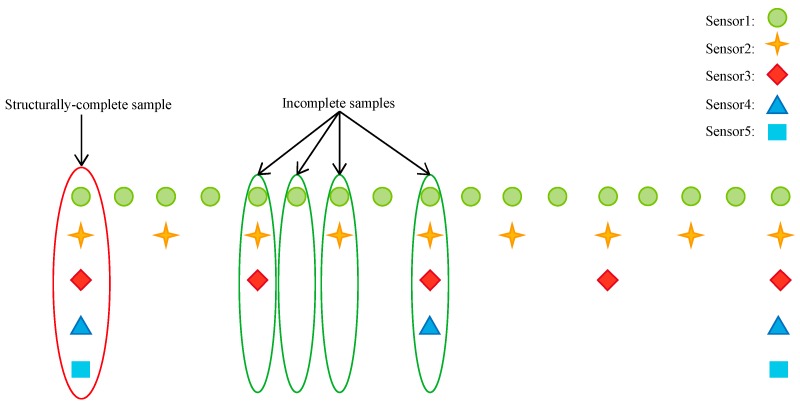
Different sampling rates of sensors.

In practical engineering, the sampling rates of different sensors may be different, which will lead to a rather small number of samples with a complete structure. For example, there are five sensors with different sampling rates in Figure 1. The sampling rate of sensor 1 is two times that of sensor 2, four times that of sensor 3, eight times that of sensor 4 and sixteen times that of sensor 5. Thus, only 6.25% of the samples are structurally complete while 93.75% of the samples are incomplete. Considering that only structurally complete samples can be applied as the input of the DNN, the structurally complete sample size is too small to train an accurate fault diagnosis model. To address this problem, this paper presents a fault diagnosis framework of missing data based on transfer learning, which makes full use of a large number of structurally incomplete samples. To introduce the proposed framework comprehensively and systematically, this section is divided into three parts as follows: transfer from the fault diagnosis model of missing data to the model of structurally complete data, transfer from the fault diagnosis model of structurally complete data to the model of missing data and the real-time online diagnosis of multi-rate sampling data.

### 3.1. Transfer from Fault Diagnosis Model of Missing Data to the Model of Structurally Complete Data

**Figure 2:**
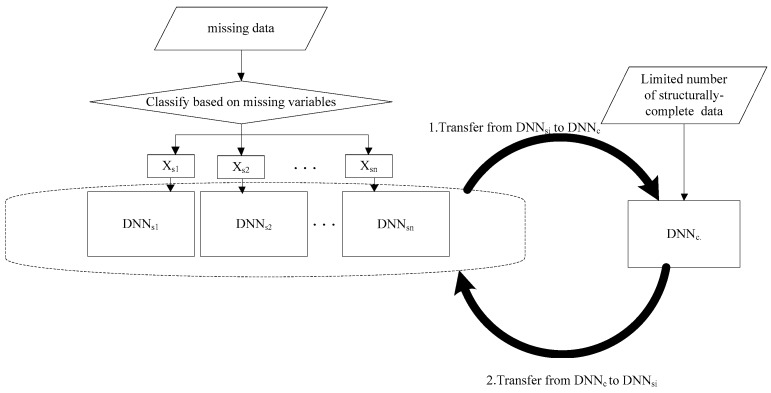
The fault diagnosis framework with missing data.

Samples with incomplete structures may lose some information but contain other useful information. It is necessary to migrate them to a structurally complete fault diagnosis model. This section explains how to learn and extract fault features from a huge number of incomplete data and then migrate these extracted features from an incomplete data model to a structurally complete data model to enhance the fault diagnosis accuracy of the latter model. The fault diagnosis framework with missing data is shown in Figure 2. The algorithm is as follows:

Step 1: Classifying samples based on missing data.

The sample set is divided into complete sample set $X_{c}$ and incomplete sample set which is further classified into n categories $X_{s1,}X_{s2,}\ldots X_{sn}$. Taking Figure 1 as an example, the samples are divided into five categories, among which $X_{c}$ is structurally complete sample set while $X_{s1,}X_{s2,}X_{s3}$ and $X_{\mathrm{sn}}$ are structurally incomplete sample sets. When the sample value of sensor 5 is unavailable, $X_{s1}$ is used to represent the missing data set. When the sample values of sensor 4 and 5 are unavailable, $X_{s2}$ is used to represent the missing data set. When the sample values of sensor 3, 4 and 5 are unavailable, $X_{s3}$ is used to represent the missing data set. When the sample values of sensor 2, 3, 4 and 5 are unavailable, $X_{sn}$ is used to represent the missing data set. Supposing a structurally complete sample contains *P* variables, $C_{pm}(x_{m})$ is 0 if the *p*th variable of sample $x_{m}$ is missing and 1 otherwise as indicated in Equation (13). $C_{m}(x_{m})$ thus represents the missing state of $x_{m}$ as shown in Equation (14).

(13) $$C_{pm}(x_{m})=\left\{ \begin{array}{l} 1(x_{pm}\neq NAN)\\ 0(x_{pm}=NAN) \end{array}\right.,p\in \{1,2,\ldots ,P\}$$

(14) $$C_{m}(x_{m})=C_{1m}(x_{m})C_{2m}(x_{m})\ldots C_{Pm}(x_{m})$$

(15) $$C_{m}(x_{m})=\underset{P}{\underset{︸}{11\ldots 1}}$$

(16) $$\exists p\in \left\{1,2,\ldots ,P\right\},\;C_{pm}(x_{m})=0$$

(17) $$\begin{array}{l} x_{i},x_{j}\in X_{q}\Leftrightarrow \left\{ \begin{array}{l} C_{1i}(x_{i})=C_{1j}(x_{j})\\ C_{2i}(x_{i})=C_{2j}(x_{j})\\ \vdots \\ C_{Pi}(x_{i})=C_{Pj}(x_{j}) \end{array}\right.,q\in \{s1,s2,\ldots ,sn,c\} \end{array}$$

If Equation (15) is true, it represents that sample $x_{m}$ is complete data while that Equation (16) is true means sample $x_{m}$ is missing data. If $C_{pi}(x_{i})=C_{pj}(x_{j}),\forall p\in \{1,2,\ldots ,P\}$ is true, sample $x_{i}$ and sample $x_{j}$ either are both complete data or belong to the same type of missing data as shown in Equation (17).

Step 2: Building fault diagnosis models $DNN_{s}$ for each type of missing data.

Let us take type i as an example and build its fault diagnosis model $DNN_{si}=Feedforward(\theta_{m1},\theta_{m2},\cdots \theta_{mN};h_{m1},h_{m2},\cdots ,h_{mN}{;X}_{si})$. $DNN_{si}$ is composed by stacking N autoencoders. $h_{m1},h_{m2},\cdots ,h_{mN}$ are the neuron number of 1st, 2nd,…Nth hidden layers in $DNN_{si}$, respectively. $\theta_{mi}=\{W_{mi},b_{mi}\}$ is the set of weight matrix and bias between input layer and hidden layer of $AE_{i}$ in $DNN_{si}$ respectively and is initialized randomly. Then $\tilde{\theta_{mi}}=\{\tilde{W_{mi}},d_{mi}\}$ is the set of weight matrix and bias between hidden layer and output layer of $AE_{i}$ in $DNN_{si}$ respectively and is randomly initialized as well. Likewise, we can build fault diagnosis models $DNN_{s1,}DNN_{s2,}\ldots ,DNN_{sn}$ for missing data types 1, 2,…, *n* respectively.

Step 3: Training the models $DNN_{s1,}DNN_{s2,}\ldots ,DNN_{sn}$.

Taking $DNN_{si}$ as an example, $DNN_{si}$ is trained by historical missing data set $X_{si}$ and obtain the feature shown in $H_{mN}=\sigma (W_{mN}\cdots (\sigma (W_{m2}(\sigma (W_{m1}X_{si}+b_{m1})+b_{m2}))+\cdots b_{mN})$. The model updates parameters $\theta_{mj}$ and $\tilde{\theta_{mj}}$ by Equations (5)–(8).

Step 4: Optimizing the models $DNN_{s1,}DNN_{s2,}\ldots ,DNN_{sn}$.

$H_{mN}$ is used as input data to train Softmax model. Still taking $DNN_{si}$ as an example, $DNN_{si}$ is optimized by backpropagation algorithm and parameters $\theta_{ms}$ are updated.

Step 5: Implementing transfer based on multi-rate sampling and building fault diagnosis model $DNN_{c}$ of complete data.

**Figure 3:**
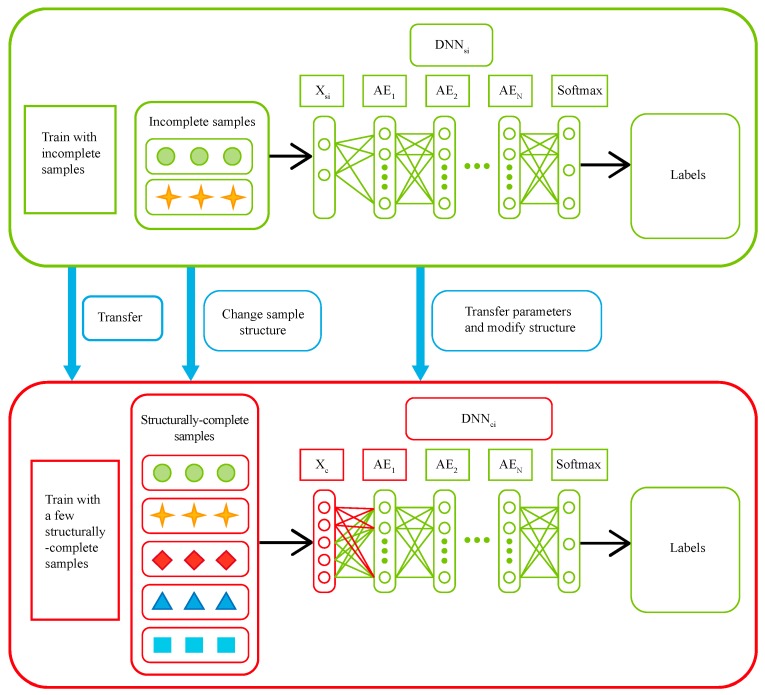
The transfer process of fault diagnosis model for multi-rate sampling.

Although missing data is incomplete, it still contains fault information. We thus extract fault features from incomplete samples and transfer the extracted features to the structurally complete model to improve the fault diagnosis accuracy of the model. The transfer can cause the structure of DNN to be modified due to the fact that incomplete and complete samples are different in dimension while the input layer size of the first layer should be the same as the dimensionalities of samples. This transfer process is illustrated in Figure 3. $DNN_{s}$ for every type of missing data is transferred to $DNN_{c}$ respectively. $DNN_{ci}$ represents the model obtained through $DNN_{si}$ migration. Let us take the migration from $DNN_{si}$ to $DNN_{ci}$ as an example. At first, the system checks every variable to see if it is missing in $DNN_{si}$. If the variable is not missing, then the encoding parameter $\theta {\;}_{m1}$ of the first layer in $DNN_{si}$ can be migrated to the input layer of $DNN_{ci}$. Otherwise the corresponding parameter of the first layer in $DNN_{ci}$ is randomly initialized as shown in Equations (18)–(20). It is assumed that structurally-complete data has *P* variables while incomplete data has l variables. Without loss of generality, the l variables of incomplete data can be assumed to be the first l variables of structurally-complete data. r represents a random number. The encoding parameters of the remaining layers in $DNN_{si}$ are migrated to $DNN_{ci}$ in Equation (21). The decoding parameters $\tilde{\theta {\;}_{m1}}$ of the first layer in $DNN_{si}$ can be similarly migrated to $DNN_{ci}$ as shown in Equations (22)–(24). Last but not least, the decoding parameters of the remaining layers in $DNN_{si}$ are migrated to $DNN_{ci}$ as indicated in Equation (25).

(18) $$\forall x\in X_{si},\left\{ \begin{array}{l} \theta_{c1}(:,j)=\theta_{m1}(:,j)\;\mathrm{if}C_{j}(x)=1\\ \theta_{c1}(:,j)=rand()\;\mathrm{if}C_{j}(x)=0 \end{array}\right.$$

(19) $$\theta_{m1}=\left[\begin{array}{ccccc} w_{11} & w_{12} & \ldots & w_{1l} & b_{1}\\ w_{21} & w_{22} & \ldots & w_{2l} & b_{2}\\ \vdots & \vdots & \vdots & \vdots & \vdots \\ w_{t1} & w_{t2} & \ldots & w_{tl} & b_{t} \end{array}\right]$$

(20) $$\theta_{c1}=\left[\begin{array}{ccccccccc} w_{11} & w_{12} & \ldots & w_{1l} & r_{1(l+1)} & r_{1(l+2)} & \ldots & r_{1P} & b_{1}\\ w_{21} & w_{22} & \ldots & w_{2l} & r_{2(l+1)} & r_{2(l+2)} & \ldots & r_{2P} & b_{2}\\ \vdots & \vdots & \vdots & \vdots & \vdots & \vdots & \vdots & \vdots & \vdots \\ w_{t1} & w_{t2} & \ldots & w_{tl} & r_{t(l+1)} & r_{t(l+2)} & \ldots & r_{tP} & b_{t} \end{array}\right]$$

(21) $$\begin{array}{c} \theta_{c2}=\theta_{m2}\\ \vdots \\ \theta_{cN}=\theta_{mN} \end{array}$$

(22) $$\forall x\in X_{si},\left\{ \begin{array}{l} \tilde{\theta_{c1}}(j,:)=\tilde{\theta_{m1}}(j,:)\;\mathrm{if}C_{j}(x)=1\\ \tilde{\theta_{c1}}(j,:)=rand()\;\mathrm{if}C_{j}(x)=0 \end{array}\right.$$

(23) $$\tilde{\theta_{m1}}=\left[\begin{array}{ccccc} \tilde{w_{11}} & \tilde{w_{12}} & \ldots & \tilde{w_{1t}} & d_{1}\\ \tilde{w_{21}} & \tilde{w_{22}} & \ldots & \tilde{w_{2t}} & d_{2}\\ \vdots & \vdots & \vdots & \vdots & \vdots \\ \tilde{w_{l1}} & \tilde{w_{l2}} & \ldots & \tilde{w_{lt}} & d_{l} \end{array}\right]$$

(24) $$\tilde{\theta_{c1}}=\left[\begin{array}{ccccc} \tilde{w_{11}} & \tilde{w_{12}} & \ldots & \tilde{w_{1t}} & d_{1}\\ \tilde{w_{21}} & \tilde{w_{22}} & \ldots & \tilde{w_{2t}} & d_{2}\\ \vdots & \vdots & \vdots & \vdots & \vdots \\ \tilde{w_{l1}} & \tilde{w_{l2}} & \ldots & \tilde{w_{lt}} & d_{l}\\ r_{(l+1)1}^{} & r_{(l+1)2}^{} & \ldots & r_{(l+1)t} & r_{l+1}\\ r_{(l+2)1} & r_{(l+2)2} & \ldots & r_{(l+2)t} & r_{l+2}\\ \vdots & \vdots & \vdots & \vdots & \vdots \\ r_{P1} & r_{P2} & \ldots & r_{Pt} & r_{P} \end{array}\right]$$

(25) $$\begin{array}{c} \tilde{\theta_{c2}}=\tilde{\theta_{m2}}\\ \vdots \\ \tilde{\theta_{cN}}=\tilde{\theta_{mN}} \end{array}$$

Therefore, we build fault diagnosis model of complete data by transfer learning $DNN_{ci}=Feedforward(\theta_{c1},\theta_{c2},\cdots \theta_{cN};h_{c1},h_{c2},\cdots h_{cN}{;X}_{c})$. $DNN_{c1},DNN_{c2},\ldots ,DNN_{cn}$ are also built in the similar way to $DNN_{ci}$.

Step 6: Training the models $DNN_{c1},DNN_{c2},\ldots ,DNN_{cn}$.

Still taking $DNN_{ci}$ as an example, $DNN_{ci}$ is trained by structural complete data set $X_{c}$ and features are obtained by $H_{cN}=\sigma (W_{cN}\cdots (\sigma (W_{c2}(\sigma (W_{c1}X_{c}+b_{c1})+b_{c2}))+\cdots b_{cN})$. The model updates parameters $\theta_{cj}$ by Equations (5) and (6).

Step 7: Optimizing the models $DNN_{c1},DNN_{c2},\ldots ,DNN_{cn}$.

$H_{cN}$ is applied to train Softmax model as input data and $DNN_{c1},DNN_{c2},\ldots ,DNN_{cn}$ are optimized by backpropagation algorithm respectively.

### 3.2. Transfer from Fault Diagnosis Model of Structurally-Complete Data to the Model of Missing Data

Although the number of structurally complete samples is small, structurally complete samples contain all the information monitored. Therefore, the structurally complete fault diagnosis model can be migrated to structurally incomplete fault diagnosis models. From all the models $DNN_{c1},DNN_{c2},\ldots ,DNN_{cn}$, we select one with the highest accuracy which is assumed to be $DNN_{ci}$. Then by training and optimizing the model $DNN_{ci}$ repeatedly, a better $DNN_{ci}$ model can be obtained and in turn migrated to $DNN_{s1,}DNN_{s2,}\ldots ,DNN_{sn}$.

Step 1: Transferring from fault diagnosis model of structurally-complete data to the model of missing data.

Structurally-complete data contains the overall information of the fault despite the fact that the sample size is limited. Therefore, complete samples can also be made full use of to extract fault features which are then transferred to the model of incomplete data for a higher accuracy of fault diagnosis. According to the dimensionalities of complete and incomplete samples, the input layer size of $DNN_{ci}$ is more than $DNN_{s}$. We use the migration from $DNN_{ci}$ to $DNN_{si}$ as an example. Firstly, the system checks $DNN_{si}$ for missing any variables. Secondly, the encoding parameters $\theta {\;}_{c1}$ of the first layer in $DNN_{ci}$ can be migrated to the input layer of $DNN_{si}$ as indicated in Equation (26). Under the assumption that structurally-complete data has *P* variables while incomplete data has l variables, the l variables of incomplete data can be considered as the first l variables of structurally-complete data without loss of generality. This is shown in Equations (27) and (28). Thirdly, the decoding parameters $\tilde{\theta {\;}_{c1}}$ of the first layer in $DNN_{ci}$ can be migrated to $DNN_{si}$ in Equation (30)–(32). Fourthly, the encoding and decoding parameters of the remaining layers in $DNN_{ci}$ can be migrated to $DNN_{si}$ as indicated in Equations (29) and (33).

(26) $$\theta_{m1}(:,j)=\theta_{c1}(:,j)\;\mathrm{if}C_{j}(x)=1,\forall x\in X_{si}$$

(27) $$\theta_{c1}=\left[\begin{array}{ccccccccc} w_{11} & w_{12} & \ldots & w_{1l} & w_{1(l+1)} & w_{1(l+2)} & \ldots & w_{1P} & b_{1}\\ w_{21} & w_{22} & \ldots & w_{2l} & w_{2(l+1)} & w_{2(l+2)} & \ldots & w_{2P} & b_{2}\\ \vdots & \vdots & \vdots & \vdots & \vdots & \vdots & \vdots & \vdots & \vdots \\ w_{t1} & w_{t2} & \ldots & w_{tl} & w_{t(l+1)} & w_{t(l+2)} & \ldots & w_{tP} & b_{t} \end{array}\right]$$

(28) $$\theta_{m1}=\left[\begin{array}{ccccc} w_{11} & w_{12} & \ldots & w_{1l} & b_{1}\\ w_{21} & w_{22} & \ldots & w_{2l} & b_{2}\\ \vdots & \vdots & \vdots & \vdots & \vdots \\ w_{t1} & w_{t2} & \ldots & w_{tl} & b_{t} \end{array}\right]$$

(29) $$\begin{array}{c} \theta_{m2}=\theta_{c2}\\ \vdots \\ \theta_{mN}=\theta_{cN} \end{array}$$

(30) $$\tilde{\theta_{m1}}(j,:)=\tilde{\theta_{c1}}(j,:)\;\mathrm{if}C_{j}(x)=1,\forall x\in X_{si}$$

(31) $$\tilde{\theta_{c1}}=\left[\begin{array}{ccccc} \tilde{w_{11}} & \tilde{w_{12}} & \ldots & \tilde{w_{1t}} & d_{1}\\ \tilde{w_{21}} & \tilde{w_{22}} & \ldots & \tilde{w_{2t}} & d_{2}\\ \vdots & \vdots & \vdots & \vdots & \vdots \\ \tilde{w_{l1}} & \tilde{w_{l2}} & \ldots & \tilde{w_{lt}} & d_{l}\\ w_{(l+1)1}^{} & w_{(l+1)2}^{} & \ldots & w_{(l+1)t} & d_{l+1}\\ w_{(l+2)1} & w_{(l+2)2} & \ldots & w_{(l+2)t} & d_{l+2}\\ \vdots & \vdots & \vdots & \vdots & \vdots \\ w_{P1} & w_{P2} & \ldots & w_{Pt} & d_{P} \end{array}\right]$$

(32) $$\tilde{\theta_{m1}}=\left[\begin{array}{ccccc} \tilde{w_{11}} & \tilde{w_{12}} & \ldots & \tilde{w_{1t}} & d_{1}\\ \tilde{w_{21}} & \tilde{w_{22}} & \ldots & \tilde{w_{2t}} & d_{2}\\ \vdots & \vdots & \vdots & \vdots & \vdots \\ \tilde{w_{l1}} & \tilde{w_{l2}} & \ldots & \tilde{w_{lt}} & d_{l} \end{array}\right]$$

(33) $$\begin{array}{c} \tilde{\theta_{m2}}=\tilde{\theta_{c2}}\\ \vdots \\ \tilde{\theta_{mN}}=\tilde{\theta_{cN}} \end{array}$$

Step 2: Building fault diagnosis models $DNN_{s}$ for each type of missing data.

Let us take type i as an example and build its fault diagnosis model $DNN_{si}=Feedforward(\theta_{m1},\theta_{m2},\cdots \theta_{mN};h_{m1},h_{m2},\cdots h_{mN}{;X}_{si})$.

Step 3: Training the models $DNN_{s1,}DNN_{s2,}\ldots ,DNN_{sn}$.

Taking $DNN_{si}$ as an example, $DNN_{si}$ is trained by historical missing data set $X_{si}$ and obtained features is shown in $H_{mN}=\sigma (W_{mN}\cdots (\sigma (W_{m2}(\sigma (W_{m1}X_{si}+b_{m1})+b_{m2}))+\cdots b_{mN})$. $H_{mN}$ is used as input data to train Softmax model and the model updates parameters $\theta_{ms}$.

Step 4: Optimizing the models $DNN_{s1,}DNN_{s2,}\ldots ,DNN_{sn}$.

Using $DNN_{si}$ as an example again, $DNN_{si}$ is optimized by backpropagation algorithm. In this way, $DNN_{ci}$ and $DNN_{si}$ are trained alternately until a satisfactory accuracy of fault diagnosis is achieved.

### 3.3. Online Diagnosis of Multi-Rate Sampling Data

The framework proposed in this paper can carry out real-time online fault diagnosis for missing data. Because incomplete data may disappear different values of different sensors, incomplete data can be divided into a variety of missing data types. In the offline phase, a corresponding fault diagnosis model is established for each missing data type according to the algorithm in Section 3.1 and Section 3.2. In the online diagnosis stage, the first step judges whether the data $x_{online}(t)$ at time t will be missing data or complete data.

Step 1: Judge whether the data $x_{online}(t)$ at time t will be missing data or complete data.

If $x_{online}(t)$ is structurally-complete data and has P values according to Equation (34), go to step 4. Otherwise move to step 2.

(34) $$\begin{array}{l} C(x_{online}(t))=\underset{P}{\underset{︸}{11\ldots 1}}\Leftrightarrow x_{online}(t)\in X_{c}\\ \exists C_{p}(x_{online}(t))=0,\forall p\in \left\{1,2,\ldots ,P\right\}\Leftrightarrow x_{online}(t)\notin X_{c} \end{array}$$

Step 2: Judge the type of missing data according to Equation (35).

(35) $$\exists i\in \{s1,s2,\ldots ,sn\},C(X_{i})=C(x_{online}(t))\Rightarrow x_{online}(t)\in X_{i}$$

Step 3: Fault diagnosis is carried out by using the corresponding $DNN_{si}$ and the model is updated. Turn to step 5.

Step 4: Fault diagnosis is carried out by using the model $DNN_{ci}$ and the model is updated.

**Figure 4:**
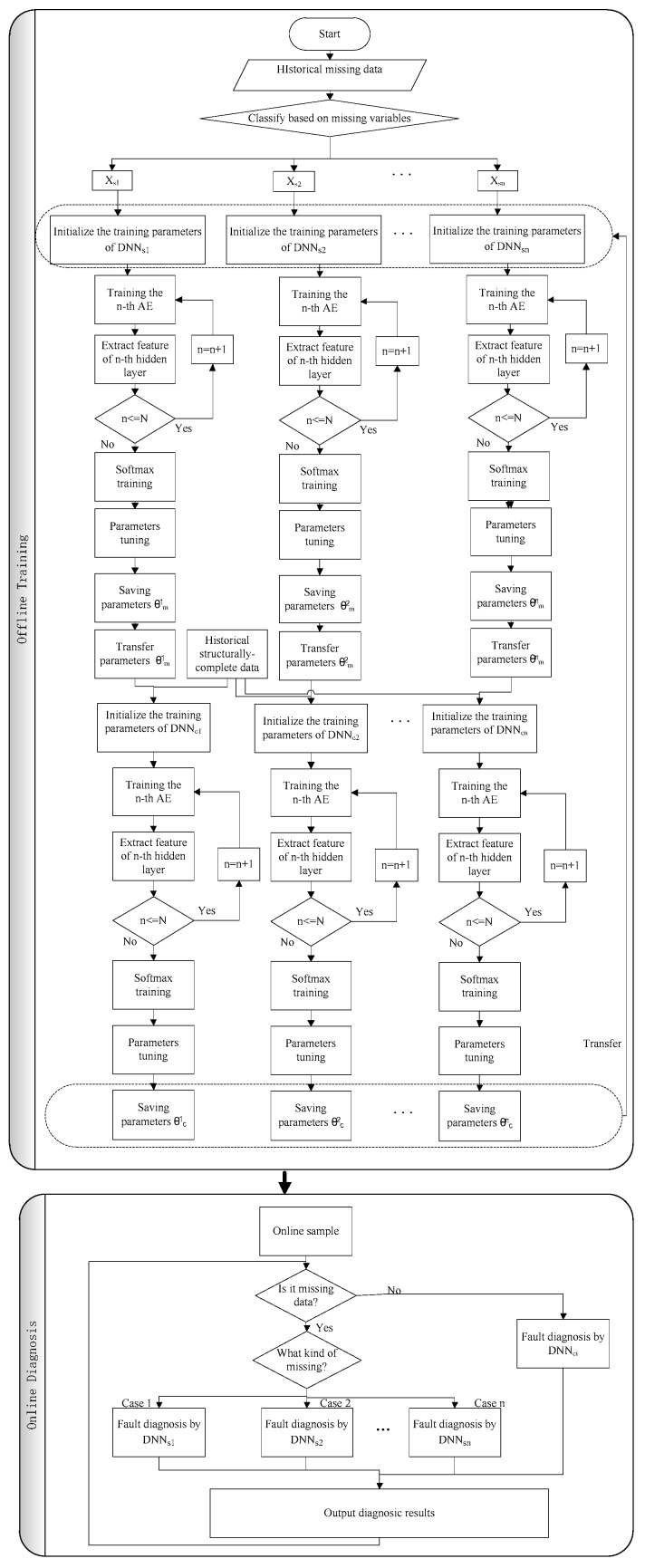
Fault diagnosis flowchart with missing data based on transfer learning.

Step 5: Output diagnostic results and wait the next data. Turn to step 1. Fault diagnosis flowchart based on transfer learning is shown in Figure 4.

## 4. Experiment and Analysis

### 4.1. Experiment Platform

The paper applies a QPZZ-II rotating machinery vibration experimental platform system, which can simulate gear fault [48]. The main parameters include: a maximum speed of 1470 r/min, three wheels (normal, pit and worn tooth) and two pinions (normal, worn). In this experiment, wheels and pinions are used as experimental objects. Rotational speed is 1470 r/min. Nine sensors are employed to collect information as shown in Table 2. In this experiment, the complete structure of the sample includes 9 variables collected from 9 sensors. Let’s assume that the sampling rates of sensors 1, 3, 5 and 7 are two times that of sensor 6, four times that of sensor 4, eight times that of sensor 2 and sixteen times that of sensor 8 and 9. Thus, only 6.25% of the samples are structurally complete while 93.75% of the samples are incomplete. There are 5 healthy states of the gearbox and details are indicated in Table 3.

### 4.2. Transfer from Missing Data Model to Structurally-Complete Model

In order to validate the effectiveness of the proposed framework, experiments are carried out with 2 missing variables, 3 missing variables, 4 missing variables and 5 missing variables, respectively, and details are shown in Table 4. Taking incomplete data with 4 variables as an example, the incomplete data obtains four variables from sensors and the remaining five variables are missing corresponding to the sensors 2, 4, 6, 8 and 9 in Table 2, respectively.

The DNN represents a traditional deep neural network while the deep transfer network (DTN) represents a deep neural network with transfer learning. This paper employs a stacked autoencoder to build the DNN model. DNN has a stacked autoencoder and the Softmax layer. The stacked autoencoder consists of four autoencoders. The values of training parameters and structure parameters of DNN and DTN are listed in Table 5. To simplify the description, details of the models are shown in Table 6.

**Remark** **1.** *Different network parameters have different diagnostic results. On the one hand, this paper applied trial-and-error method to find the optimal values of parameters which are listed in Table 5. No matter which group of network parameters is used, on the other hand, experimental results can clearly show the effect of the method proposed in the paper is better than non-migration methods.*

**Figure 5:**
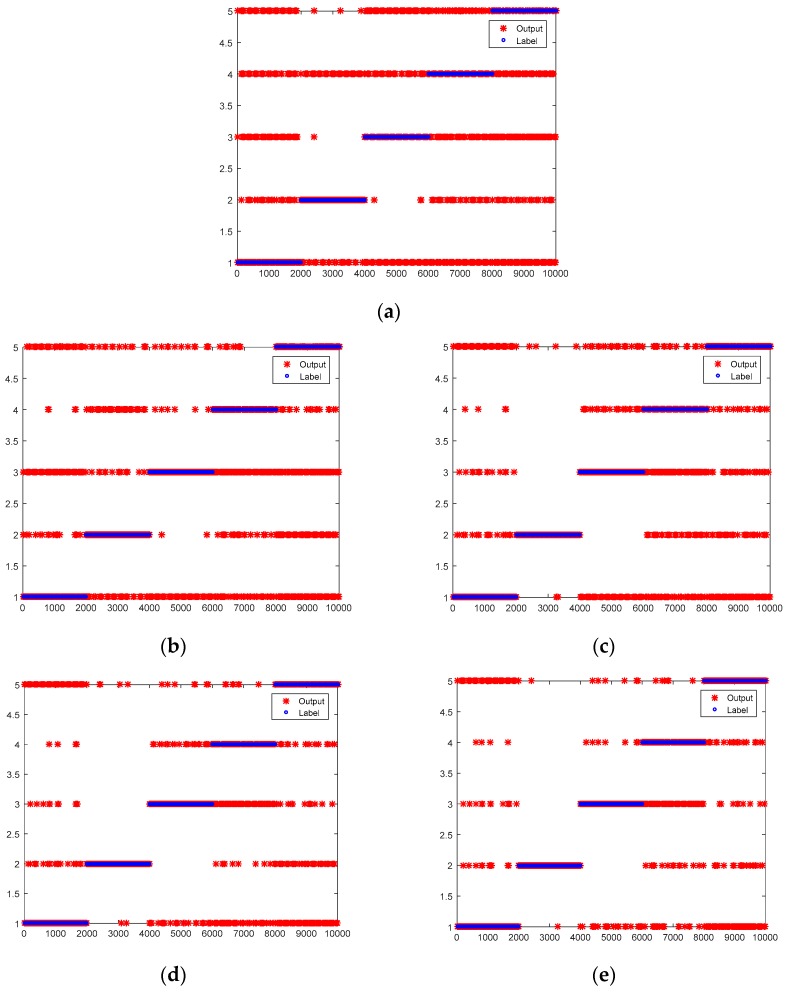
Test results of DNN and DTNs when the ratio of incomplete data to structurally-complete data is 60:1. (**a**) CDNN; (**b**) MCDTN1; (**c**) MCDTN2; (**d**) MCDTN3; (**e**) MCDTN4.

In order to verify the effectiveness of this method, experiments are carried out at an incomplete data to structurally-complete data ratio of 60:1, 30:1 and 20:1 respectively. The results are shown in Table 7 and Figure 5 when the ratio of incomplete data to structurally-complete data is 60:1. Incomplete data has 600 samples for each type as training data. Structurally-complete data has 10 samples for each type as training data. Test data for structurally-complete and incomplete data has 2000 samples for each type respectively. The red star denotes actual output while the blue circle denotes the label in Figure 5. The first 2000 samples represent normal data recorded as label 1 and the second 2000 samples represent wheel pit recorded as label 2, and so forth. As the results indicate, the average accuracy of DNN is 41.55% while the average accuracy of DTNs are 65.64%, 67.57%, 73.48% and 78.31% respectively. DTNs are at least 24.09% higher on average than DNN. For all labels, the classification accuracies of DTNs perform significantly better than DNN. Especially for label 3, the accuracy of DTNs are at least 48.80% higher than DNN. The average accuracy of MCDTN4 is higher than MCDTN3. The average accuracy of MCDTN3 is higher than MCDTN2. Similarly, the average accuracy of MCDTN2 is higher than MCDTN1. The more information the data has, the better the effect after migration.

When the ratio of missing data to structurally-complete data is 30:1, the results are indicated in Table 8. Incomplete data have 600 samples for each type as training data. Structurally-complete data have 20 samples for each type as training data. Test data for structurally-complete and incomplete data have 2000 samples for each type, respectively. Results show that the accuracy of DNN averages 53.89% while the average accuracy of DTNs are 68.58%, 73.78%, 77.67% and 82.64% respectively. On average, DTNs have at least 14.69% higher classification accuracy compared with DNN. For all labels, DTNs are higher in accuracy than DNN. For label 3, the accuracy of DTNs are at least 31.40% higher than DNN. Especially for label 2, the lowest accuracy of DTNs is 98.85% and the accuracy of DNN is 69.20%. The results of Table 8 show that MCDTN4 has the highest average accuracy, followed by MCDTN3, MCDTN2 and MCDTN1 in a descending order. It is consistent with the results of Table 7. But compared with Table 7, the average accuracy of the corresponding columns in Table 8 is higher than that of Table 7.

When the ratio of missing data to structurally-complete data is 20:1, the results are indicated in Table 9. The incomplete data have 600 samples for each type as training data. The structurally-complete data have 30 samples for each type as training data. Test data for structurally-complete and incomplete data have 2000 samples for each type, respectively. Results show that the accuracy of DNN averages 55.61% while the average accuracy of DTNs are 69.57%, 74.77%, 78.74% and 84.97% respectively. For all labels, DTNs are higher in accuracy than DNN. Especially for label 4, the accuracy of MCDTN4 is as high as 90.40% and the accuracy of CDNN is 35.00%. Similar to Table 7 and Table 8, the average accuracy of MCDTN4 is higher than the other three networks, following which MCDTN3 is the second and MCDTN2 is the third. MCDTN1 has the lowest average accuracy. As the number of complete samples increases, the average accuracy of the corresponding columns in Table 9 is higher than that of Table 8 and the average accuracy of the corresponding columns in Table 8 is higher than that of Table 7.

### 4.3. Transfer from Structurally-Complete Model to Missing Data Model

The accuracy of the fault diagnosis model based on missing data is not high because the missing data is incomplete. However, a well-trained fault diagnosis model of structurally complete data in the offline phase can in turn be used to diagnose the missing data. Through several migrations, training and optimization, a better structurally complete model can be obtained and in turn migrated to missing data. Incomplete data have 600 samples for each type. Test data of structurally-complete and missing data have 2000 samples for each type, respectively. Incomplete data model without transfer learning is only trained by incomplete data. A trained fault diagnosis model of structurally-complete data is transferred to an incomplete data model, in which we can get an incomplete data model with transfer learning.

The results are shown in Table 10. From the results, we can find that the average accuracy of incomplete data model with transfer learning is always higher than corresponding model without transfer learning. The accuracy of CMDTN3 reaches 95.19% and is 5.85% higher than MDNN3. 

### 4.4. Online Diagnosis of Multi-Rate Sampling Data

**Figure 6:**
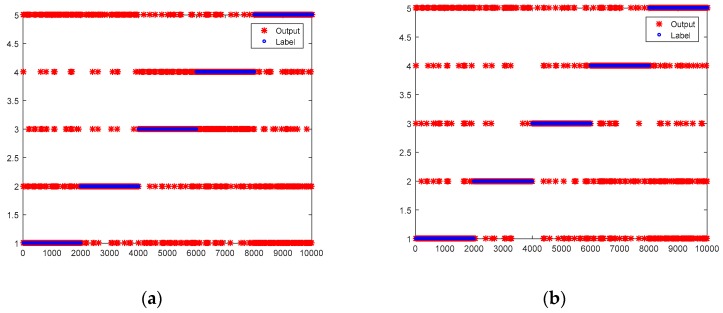
Test results of online diagnosis models without and with transfer learning. (**a**) Online diagnosis models without transfer learning; (**b**) Online diagnosis models with transfer learning.

The structurally complete DNN model obtained in Section 3.1 and the incomplete DNN models obtained in Section 3.2 are used in the online phase. Incomplete data with 5 missing variables, 4 missing variables, 3 missing variables and 2 missing variables accounted for 50%, 25%, 12.5% and 6.25% respectively. The results are shown in Table 11 and Figure 6. As results indicate, the average accuracy of online diagnosis models without transfer learning is 88.42% while the average accuracy of the models with transfer learning is 91.36%. For all labels, the classification accuracies of online diagnosis models with transfer learning perform better than those without transfer learning. Because incomplete data with 5 missing variables and 4 missing variables account for 75%, this result is consistent with the offline situation shown in Table 10.

### 4.5. Analysis of Time Complexity

This paper employs a stacked autoencoder to build the DNN model. The DNN has a stacked autoencoder and a Softmax layer. The stacked autoencoder consists of four autoencoders. Let us suppose that an operation takes time as 1 unit. m is the number of samples; n_i_ is the neuron number of input layer; n_1_, n_2_, n_3_ and n_4_ are the neuron number of 1st, 2nd, 3rd and 4th hidden layers respectively; s denotes the number of classifiers; l is the number of iterations; n_b_ represents batchsize; and c is the time spent in calculating gradients. As shown in Equations (36)–(39), the time complexity of AE_1_, AE_2_, AE_3_ and AE_4_ is $O(m*n_{i}*n_{1}*l_{1})$, $O(m*n_{1}*n_{2}*l_{2})$, $O(m*n_{2}*n_{3}*l_{3})$ and $O(m*n_{3}*n_{4}*l_{4})$, respectively. The time complexity of Softmax and backpropagation is $O(n_{4}*s*l_{s})$ and $O(n_{b}*c*l_{b})$, respectively. Thus, the time complexity of the traditional DNN is $O(m*n^{2}*l)$ according to Equation (40).

(36) $$O(m*(n_{i}(n_{1}+1)+n_{1}(n_{i}+1))*l_{1})=O(m*n_{i}*n_{1}*l_{1})$$

(37) $$O(m*(n_{1}(n_{2}+1)+n_{2}(n_{1}+1))*l_{2})=O(m*n_{1}*n_{2}*l_{2})$$

(38) $$O(m*(n_{2}(n_{3}+1)+n_{3}(n_{2}+1))*l_{3})=O(m*n_{2}*n_{3}*l_{3})$$

(39) $$O(m*(n_{3}(n_{4}+1)+n_{4}(n_{3}+1))*l_{4})=O(m*n_{3}*n_{4}*l_{4})$$

(40) $$O(m*n_{i}*n_{1}*l_{1}+m*n_{1}*n_{2}*l_{2}+m*n_{2}*n_{3}*l_{3}+m*n_{3}*n_{4}*l_{4}+n_{4}*s*l_{s}+n*c*l_{b})=O(m*n^{2}*l)$$

The migration algorithm is summarized in three steps: 1. training source model and extracting fault features; 2. migrating these extracted features from source model to target model; and 3. training the target model based on transfer learning. The time complexity of step 1 is the same as that of a traditional DNN. The time complexity of step 2 is O(n) and the time complexity of step 3 is the same as step 1. So the time complexity of DTN is the same as that of DNN according to Equation (41).

(41) $$O(m*n^{2}*l+n+m*n^{2}*l)=O(m*n^{2}*l)$$

In the offline phase, the running times of CDNN and MCDTNs are shown in Table 12 when the ratio of incomplete data to structurally-complete data is 20:1. By comparing the runtime, MCDTN runs about six times as long as CDNN. The time complexity of CDNN is in the same order of magnitude as that of MCDTNs. In the offline phase, the running time of MDNNs and CMDTNs is shown in Table 13. By comparing the runtime, CMDTN runs about three times as long as MDNN. The time complexity of MDNNs is in the same order of magnitude as that of CMDTNs. In the online phase, the running time of diagnosis models without transfer learning and with transfer learning for 10 000 online data are shown in Table 14. By comparing the runtime, diagnosis models with transfer learning run about the same time as diagnosis models without transfer learning.

## 5. Conclusions and Future Work

In practical engineering, the sampling rates of different sensors may be different, which will lead to a rather small number of samples with complete structure. However, a large number of samples are required to ensure the efficiency of deep learning based fault diagnosis methods. This paper therefore proposes a fault diagnosis framework of missing data based on transfer learning. The suggested framework consists of three phases: transfer from fault diagnosis model of missing data to the model of structurally complete data, transfer from fault diagnosis model of structurally complete data to the model of missing data and real-time online diagnosis of multi-rate sampling data.

The paper utilities the QPZZ-II rotating machinery vibration experimental platform system to validate the effectiveness of the proposed framework. Results of experiments indicate that structurally-complete models with transfer learning always have higher fault diagnosis accuracy than those without transfer learning for every label when the ratios of missing data to structurally-complete data are 60:1, 30:1 and 20:1 respectively. As for the transfer from the fault diagnosis model of structurally complete data to the model of missing data, every missing data model with learning is higher in accuracy than the corresponding missing data model without learning. Therefore, we come to the conclusion that the proposed fault diagnosis framework based on transfer learning can improve the accuracy of both structurally complete and missing data models. On the one hand, the framework learns the information from the missing data and migrates obtained features from the missing data model to a structurally-complete model in order to increase the fault diagnosis accuracy of the structurally complete model. On the other hand, a well-trained structurally complete model is conversely transferred to the missing data model, which can in turn help with fault diagnosis in the case of missing data.

Finally, we need to point out that there still exists the limitation of the presented framework: negative transfer can occur when the ratio of incomplete data to structurally-complete data is 10:1. Our future research, thus, will aim to design a door mechanism to filter out negative information and further enhance the accuracy of fault diagnosis model with transfer learning.

## Figures and Tables

**Table 1:** A comparison between the relevant literature and this paper.

Article	Source Task	Target Task	Method	Innovation
[46]	some labeled subject data	unlabeled subject data	domain adaptation	proposing online and offline weighted adaptation regularization algorithms to reduce classifier calibration
[47]	a group of EEG signals	another group of EEG signals	transfer learning, semi-supervised learning and TSK fuzzy system	combining TL, SSL and TSK fuzzy system models to increase the robustness, accuracy and interpretability of the EEG signal classifier
[29]	a working condition	another working condition	domain adaptation	the first application of domain adaptation to fault diagnosis
[30]	a working condition	another working condition	joint distribution adaptation	presenting a fault diagnosis framework with joint distribution adaptation
[31]	nature images	gearbox fault data	feature migration	introducing a deep convolutional neural network-based transfer learning approach to deep feature extraction
[32]	a working condition	another working condition	feature migration	presenting a transfer learning method based on neural networks for fault diagnosis of rolling bearings
This paper	incomplete data	structurally complete data	feature migration	proposing a fault diagnosis framework of missing data based on transfer learning

**Table 2:** Details of sensors equipped on the gearbox.

Sequence Number	Sensor
1	rotate speed of photoelectric Sensor
2	X direction displacement of input axis
3	Y direction displacement
4	acceleration of bearing Y of the motor side of input axis
5	acceleration of bearing Y of the motor side of output axis
6	acceleration of bearing Y of the load side of input axis
7	acceleration of bearing Y of the load side of output axis
8	acceleration of bearing X of the load side of output axis
9	magneto electric velocity of bearing X of the load side of output axis

**Table 3:** Healthy states of the gearbox.

Labels	Sensor
1	normal condition
2	wheel pit
3	wheel worn tooth
4	wheel worn tooth and pinion worn
5	wheel pit and pinion worn

**Table 4:** Details of missing data.

The Number of Variables Contained in Missing Data	Missing Variables
1	2,4,6,8,9
2	2,4,8,9
3	2,8,9
4	8,9

**Table 5:** The values of deep neural network (DNN) and deep transfer network (DTN) parameters.

Parameter	Value
The neuron number of 1st hidden layer	100
The neuron number of 2nd hidden layer	200
The neuron number of 3rd hidden layer	101
The neuron number of 4th hidden layer	50
Iterative number	1000
Momentum coefficient	0.05
Learning rate	0.1

**Table 6:** The names and explanations of the models.

Name	Explanation
CDNN	structurally-complete DNN
MCDTN1	DTN which incomplete data with 5 missing variables is transferred to structurally-complete model
MCDTN2	DTN which incomplete data with 4 missing variables is transferred to structurally-complete model
MCDTN3	DTN which incomplete data with 3 missing variables is transferred to structurally-complete model
MCDTN4	DTN which incomplete data with 2 missing variables is transferred to structurally-complete model
MDNN1	Incomplete DNN with 5 missing variables
MDNN2	Incomplete DNN with 4 missing variables
MDNN3	Incomplete DNN with 3 missing variables
MDNN4	Incomplete DNN with 2 missing variables
CMDTN1	DTN which structurally-complete model is transferred to incomplete DNN with 5 missing variables
CMDTN2	DTN which structurally-complete model is transferred to incomplete DNN with 4 missing variables
CMDTN3	DTN which structurally-complete model is transferred to incomplete DNN with 3 missing variables
CMDTN4	DTN which structurally-complete model is transferred to incomplete DNN with 2 missing variables

**Table 7:** The accuracy of DNN and DTNs when the ratio of incomplete data to structurally-complete data is 60:1.

Label	CDNN	MCDTN1	MCDTN2	MCDTN3	MCDTN4
1	60.90	64.55	71.55	74.45	77.25
2	70.25	85.95	99.40	99.55	99.90
3	22.75	79.65	71.55	89.70	95.80
4	38.25	51.25	41.90	51.65	60.05
5	15.60	46.80	53.65	52.05	58.55
Mean	41.55	65.64	67.57	73.48	78.31

**Table 8:** The accuracy of DNN and DTNs when the ratio of incomplete data to structurally-complete data is 30:1.

Label	CDNN	MCDTN1	MCDTN2	MCDTN3	MCDTN4
1	58.30	63.20	78.70	75.35	90.50
2	69.20	99.00	98.85	99.80	99.65
3	63.00	94.40	95.05	97.20	97.95
4	34.30	36.40	42.85	58.50	76.85
5	44.65	49.90	53.45	57.50	48.25
Mean	53.89	68.58	73.78	77.67	82.64

**Table 9:** The accuracy of DNN and DTNs when the ratio of incomplete data to structurally-complete data is 20:1.

Label	CDNN	MCDTN1	MCDTN2	MCDTN3	MCDTN
1	63.00	71.60	84.45	79.20	81.15
2	74.45	98.25	99.65	99.85	99.95
3	57.95	85.90	95.70	97.45	98.60
4	35.00	41.60	45.85	62.55	90.40
5	47.65	50.50	48.20	54.65	54.75
Mean	55.61	69.57	74.77	78.74	84.97

**Table 10:** The accuracy of DNNs and DTNs when transferring from structurally-complete model to missing data model.

Label	MDNN1	CMDTN1	MDNN2	CMDTN2	MDNN3	CMDTN3	MDNN4	CMDTN4
1	84.00	86.10	83.85	85.20	83.85	94.45	80.45	94.00
2	90.55	91.05	99.85	99.95	100.00	99.30	99.95	99.70
3	91.95	95.85	95.10	95.75	97.75	98.70	97.45	98.35
4	91.85	92.35	92.40	92.70	91.50	91.30	93.90	91.55
5	79.90	83.30	77.10	88.30	73.60	92.20	80.20	93.30
Mean	87.65	89.73	89.66	92.38	89.34	95.19	90.39	95.38

**Table 11:** The accuracy of online diagnosis of multi-rate sampling data without and with transfer learning.

Label	Online Diagnosis Models without Transfer Learning	Online Diagnosis Models with Transfer Learning
1	84.30	86.80
2	95.15	95.80
3	91.20	95.70
4	87.95	92.65
5	83.50	85.85
Mean	88.42	91.36

**Table 12:** The runtime of structurally-complete fault diagnosis model without and with transfer learning in offline.(unit: second).

CDNN	MCDTN1	MCDTN2	MCDTN3	MCDTN4
36.133508	182.086362	188.777025	161.713538	174.761340

**Table 13:** The runtime of incomplete data fault diagnosis model without and with transfer learning in offline. (unit: second).

MDNN1	CMDTN1	MDNN2	CMDTN2	MDNN3	CMDTN3	MDNN4	CMDTN4
104.860291	242.961147	80.353521	248.753952	69.947270	245.761715	121.263790	252.353889

**Table 14:** The runtime of online diagnosis models without and with transfer learning. (unit: second).

DNN	DTN
0.008025	0.008507
